# Directional protein secretion by the retinal pigment epithelium: roles in retinal health and the development of age-related macular degeneration

**DOI:** 10.1111/jcmm.12070

**Published:** 2013-05-11

**Authors:** Paul Kay, Yit C Yang, Luminita Paraoan

**Affiliations:** a Department of Eye and Vision Science, Institute of Ageing and Chronic Disease, University of LiverpoolLiverpool, UK; b Department of Ophthalmology, The Royal Wolverhampton NHS TrustWolverhampton, UK

**Keywords:** retina, retinal pigment epithelium, protein secretion, polarity, age-related macular degeneration

## Abstract

The structural and functional integrity of the retinal pigment epithelium (RPE) is fundamental for maintaining the function of the neuroretina. These specialized cells form a polarized monolayer that acts as the retinal–blood barrier, separating two distinct environments with highly specialized functions: photoreceptors of the neuroretina at the apical side and Bruch's membrane/highly vascularized choriocapillaris at the basal side. The polarized nature of the RPE is essential for the health of these two regions, not only in nutrient and waste transport but also in the synthesis and directional secretion of proteins required in maintaining retinal homoeostasis and function. Although multiple malfunctions within the RPE cells have been associated with development of age-related macular degeneration (AMD), the leading cause of legal blindness, clear causative processes have not yet been conclusively characterized at the molecular and cellular level. This article focuses on the involvement of directionally secreted RPE proteins in normal functioning of the retina and on the potential association of incorrect RPE protein secretion with development of AMD. Understanding the importance of RPE polarity and the correct secretion of essential structural and regulatory components emerge as critical factors for the development of novel therapeutic strategies targeting AMD.

- Introduction
- RPE polarity
- Apical secretion from the RPE
  – Matrix Metalloproteinase 2 (MMP-2) and Tissue Inhibitor of Matrix Metalloproteinase 1 (TIMP-1)
  – Hyaluronan
  – αB Crystallin
  – Pigment Epithelium-Derived Factor (PEDF)
- Basolateral secretion from the RPE
  – Fibroblast Growth Factor 5 (FGF-5)
  – Endothelin I
  – Vascular Endothelial Growth Factor (VEGF)
  – Cystatin C
- Mechanisms of protein secretion/polarized secretion
  – Basolateral sorting signals
  – Apical sorting signals
  – Consequences of impaired RPE protein secretion
- Concluding remarks

## Introduction

The RPE consists of a monolayer of cells that form the retinal–blood barrier (RBB). On either side of this cellular monolayer lie two contrasting environments that are critical for the correct functioning of the neuroretina. Immediately on the apical side of the RPE is a thin matrix known as the interphotoreceptor matrix (IPM) 1, 2. Embedded in this matrix are the highly specialized photoreceptor cells. On the basal side of the RPE lies another unique cellular support structure, the Bruch's membrane (an elastogenesis product of the RPE/choroid) as well as the fenestrated epithelium of the choriocapillaris. The role the RPE plays in separating neural and vascular tissues is similar to that of the blood–brain barrier (BBB); the RBB, however, is unique in that the RPE acts as the impermeable barrier to nutrient/waste movement, as opposed to the endothelial cells in the vessel walls, as is the case for the BBB 3.

One of the main functions of the RPE is in the delivery of nutrients from the choroid to the photoreceptor cells, whilst transporting metabolic end products, ions and excess water in the opposite direction 4–6. This function alone renders RPE a critical role in maintaining the retinal homoeostasis. However, the RPE also carries out other essential functions in visual cycle, phagocytosis of spent photoreceptor outer segments 7, 8, light absorption 7, and the expression and secretion of retinal proteins 9. Failure of the RPE to conduct any of these processes efficiently can lead to retinal degeneration, and bring about diseases such as AMD – the leading cause of legal blindness in the Western world 10. Although multiple malfunctions within the specialized cells in the retina, most importantly in the supportive RPE, have been associated with development of this disease of multifactorial origin, clear causative processes have not yet been conclusively established.

Maintenance of the structure and function of the microenvironments on either side of the RPE *via* protein secretion is the focus of this article, with specific emphasis on the importance of directional, targeted secretion of trophic/growth factors and structural/structure-related proteins.

## RPE polarity

**Fig. 1:**
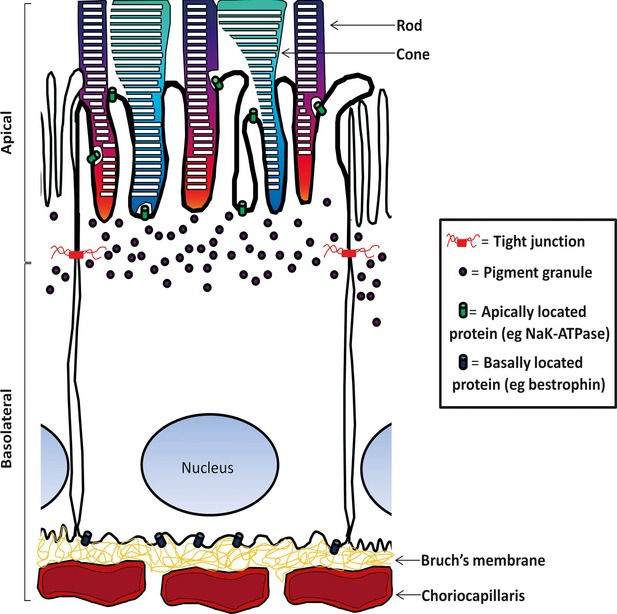
The highly polarized retinal pigment epithelium (RPE) at the interface between the retina and choroid. The organization of cellular structures into apical and basolateral domains is highlighted. At the apical surface, the microvilli of the RPE closely interact with the photoreceptor outer segments. On the basal side, the RPE are supported by the Bruch's membrane, beneath which lies the choroidal blood supply. This highly polarized arrangement ensures that the RPE remains a selectively permeable barrier between these two contrasting tissues.

The RPE displays many similarities to other epithelial layers, including a hexagonal ‘cobblestone’ appearance, organization as a single monolayer, tight junctions between cells and a highly polarized nature. Morphologically, RPE cells display polarity with apical microvilli, pigment granules and well-developed tight junctions located on the apical side of the cell, as well as basally located nuclei and membrane infolding 11, 12 (Fig. 1). A feature that distinguishes RPE from other epithelia is the fact that its apical surface does not face an acellular lumen. Instead, it is immediately adjacent to a layer of highly specialized cells, the photoreceptors.

Proteins expressed by RPE cells can also be localized to either apical or basal plasma membrane (PM) (Fig. 1), such as the apical cell membrane protein NaK-ATPase 11–13, and the basally located anion channel, bestrophin 14. The localization of such proteins can also distinguish RPE cells from other epithelia, as for example, NaK-ATPase is localized to the basal membrane in other epithelial cells 15.

The polarized organization of the RPE is crucial for its interaction with both its apical and basal side, as well as in the directionality of its protein secretion. It has been demonstrated that attainment of polarity *in vitro* increases the overall levels of growth factor secretion 16. Mechanisms by which cell polarization occur and is maintained, as well as the consequences of altered polarity and trafficking in disease states have been reviewed elsewhere 17. In the case of RPE, it is also therefore likely that factors altering the polarity of the monolayer may play an important role in the development of diseases such as AMD.

Retinal pigment epithelium cells secrete a host of growth factors and structural/structure-related proteins 9 (Table 1), and there is no doubting the importance of such secretion in supporting photoreceptor survival, as well as in maintenance of the retinal blood supply. However, the importance of directional protein secretion can often be overlooked, despite the fact it has been demonstrated that many proteins are secreted preferentially by either the apical, or basolateral PM.

**Table 1:** Proteins secreted by the RPE and their putative functions in relation to AMD

Protein	Main function/s	Links/potential links to AMD	Polarity#	References
αB Crystallin	Molecular chaperone, cytoprotection	Possible AMD biomarker, maybe involved in Drusen formation	Apical	18–20
BDNF	Neurotrophic growth factor	Protective role for the photoreceptors possibly lost during AMD	Unknown	21, 22
CFH	Inhibitor of the complement pathway	Gene variants implicated as a major AMD risk factor	Unknown	23
CNTF	Neurotrophic growth factor	May provide protection against neurodegenerative diseases, such as AMD	Unknown	21, 24
Cystatin C	Cysteine protease inhibitor	Variant B isoform associated with increased risk of “wet” AMD	Basal	25, 26
Endothelin I	Vasoconstriction/vasodilation	Mis-signalling associated with retinopathies associated with breakdown of blood–retinal barrier	Basal	27
*Fibulin 3/5	ECM protein involved in elastogenesis (fibulin 5)	Variant isoform of fibulin 5 associated with increased risk of AMD	Unknown	28, 29
FGF 2	Growth factor involved in mitogenesis, angiogenesis and cell survival	Role in choroidal neovascularization	Unknown	21, 30, 31
FGF 5	Growth factor involved in mitogenesis, angiogenesis and cell survival	Potential functional role in AMD pathophysiology	Basal	32, 33
HB-EGF	Mitogenic growth factor	Indirect role in choroidal neovascularization *via* influence on VEGF expression	Unknown	21, 34
HGF	Growth factor involved in growth, motility and morphogenesis	Provides protection to RPE cells under oxidative stress, a process frequently linked with AMD progression	Unknown	21, 35
Hyaluronan	Major component of ECM	Possible role in choroidal neovascularization *via* interaction with CD44 receptor	Apical	36, 37
IGF-I	Growth factor involved in growth and development	Role in choroidal neovascularization	Unknown	38, 39
LIF	Cytokine involved in differentiation	May aid photoreceptor survival during periods of stress, such as AMD	Unknown	21, 40
MMP-2	Zinc-dependent endopeptidase involved in ECM degradation	Activity within the Bruch's membrane altered in AMD, possible contribution to progression of “wet” AMD	Apical	41–43
MMP-9	Zinc-dependent endopeptidase involved in ECM degradation	Activity within the Bruch's membrane altered in AMD, possible contribution to progression of “wet” AMD	Unknown	41, 42
NGF	Neurotrophic growth factor	Provides protection to RPE cells under oxidative stress, a process frequently linked with AMD progression	Unknown	21, 44
PEDF	Growth factor with neurotrophic and anti-angiogenic properties	Incorrect expression/localization promotes vascularization, loss of photoreceptor support role in late-stage AMD	Apical	11, 12, 45, 46
TGF-β	Growth factor involved in proliferation and differentiation	Can cause senescence-associated changes in RPE, a process associated with early AMD	Apical	24, 47, 48
TIMP-I	Inhibitor of matrix metalloproteinases	Activity of target molecules (MMP's) altered in AMD, possible contribution to progression of “wet” AMD	Apical	41–43
Tropoelastin	Involved in formation of elastin fibres (such as in Bruch's membrane)	Potential functional role in AMD-related changes in the Bruch's membrane	Unknown	49
VEGF	Angiogenic growth factor	Incorrect expression/localization promotes vascularization role in late-stage AMD	Basal	12, 50

# Putative polarity of secretion is indicated based on experimental data published to date.

† Fibulin 3 (EFEMP 1) is known to be secreted from RPE cells 28, but its function and possible role in AMD development have not been fully characterized. The closely related Fibulin 5 is associated with AMD development and although secretion from RPE cells has not been demonstrated experimentally, it is actively secreted by other cell lines 29.

Some examples of directional protein secretion from the RPE are discussed below. The function/dysfunction of the majority of proteins presented herein have direct links to AMD pathogenesis. For those that have not, to date, been directly linked with AMD, we hypothesize how their incorrect localization/function may lead to retinal degeneration based on their involvement in fundamental biological processes and on similarities of their mechanism of action with that of known molecular determinants of the disease.

## Apical secretion from the RPE

### Matrix metalloproteinase 2 (MMP-2) and tissue inhibitor of matrix metalloproteinase 1 (TIMP-1)

Matrix metalloproteinase and TIMPs are apically secreted by the RPE 43. MMPs play a crucial role in the extracellular matrix (ECM) turnover throughout the body. Their activity is normally tightly regulated at several levels, including functional inhibition by TIMPs. It has been suggested that apically secreted MMPs/TIMPs could play a crucial role in the turnover and structural maintenance of the IPM, or possibly be involved in degrading the tips of photoreceptor outer segments, signalling their readiness for phagocytosis by the RPE 43.

Matrix metalloproteinase and TIMPs (including MMP-2 and TIMP-1) are also present basally to the RPE, in the Bruch's membrane 51–53. This indicates potential basolateral secretion of MMPs 54, 55. It is possible that the RPE is able to secrete certain MMPs/TIMPs in opposite directions depending on external cues such as cytokine stimulation 55, or signals from the ECM 43. Alternatively, MMPs/TIMPs present in Bruch's membrane could be secreted by choroidal cells. MMP activity is disturbed in the Bruch's membrane in AMD 42, and the MMPs role in angiogenesis 56 could also contribute to symptoms associated with the ‘wet’ (exudative) form of the disease. Malfunctions in controlled, directional secretion of MMPs/TIMPs could disrupt the balance of proteolytic activity on either side of the RPE, contributing to age-related changes in the IPM and Bruch's membrane.

### Hyaluronan

Hyaluronan is a widely distributed glycosaminoglycan, and is a major component of the ECM. It is secreted from the apical side of the RPE 37, where it is believed to function as the primary scaffold protein in the IPM 1. Maintaining the structural integrity of the IPM is a crucial function of the RPE, and mis-localization of hyaluronan could lead to its degeneration, subsequently affecting the survival of the light sensitive cells of the outer retina. Indeed, it has been demonstrated that the IPM is abnormally distributed in an animal model for retinal degeneration 57. The interaction of hyaluronan with the receptor CD44 has been associated with choroidal neovascularization, a common symptom of wet AMD 36. Such an interaction could be the result of mis-targeting of the protein to the wrong tissue compartment.

### αB crystallin

αB crystallin is a molecular chaperone induced by stress stimuli. By suppressing protein aggregation and inhibiting the proteolytic activity of caspase 3, it is able to play a role in cytoprotection 58. αB Crystallin is secreted from the apical surface of the RPE, where it is taken up by the photoreceptor cells. Here, it inhibits the activity of caspase 3, activates the DNA repair and apoptosis-related Poly (ADP-ribose) Polymerase (PARP) and provides the photoreceptors with protection from oxidative stress 20. More recent data suggest that the protein is released from the cell inside exosomes, and that its secretion is independent of the endoplasmic reticulum (ER)/Golgi/secretory pathway 19. αB crystallin is a major component of the IPM 59, 60 and has also been linked to AMD pathogenesis, with increased expression being proposed as a possible biomarker for the disease 18. Under severe oxidative stress, the RPE barrier can become compromised, resulting in αB crystallin aggregating on the basal side of the RPE 20. In this case, not only is the protein less available to provide a survival advantage to the photoreceptors but it can also contribute to the formation of drusen deposits 18.

### Pigment epithelium-derived factor (PEDF)

The PEDF is highly expressed in the retina, where it serves as a neurotrophic factor 61 and angiogenesis inhibitor 45. Multiple studies have shown that PEDF is secreted preferentially from the apical surface of the RPE 11, 12, 46, where it provides such neurotrophic support to the photoreceptors, and maintains a non-angiogenic retinal environment 16. Low levels of PEDF below the basal surface of the RPE may aid in preventing vascularization in this compartment as well. Dysregulated expression of both PEDF and vascular endothelial growth factor (VEGF) (discussed below) plays a role in the pathogenesis of late-stage AMD 45. A delicate balance exists on either side of the RPE regarding the concentrations of these two growth factors. Disrupting this balance can promote vascularization of the retina, whilst simultaneously decreasing photoreceptor support 16.

## Basolateral secretion from the RPE

### Fibroblast growth factor 5 (FGF-5)

Fibroblast growth factor 5 is known to play a role in a range of processes including angiogenesis 62, 63, cell survival 64 and mitogenesis 65, 66. It is secreted basally from the RPE and has a possible paracrine role in choroid function, or alternatively, acts as an autocrine survival factor for RPE cells 32. Whatever its precise function in the retina, it is conceivable that its mis-regulated secretion could have detrimental effects on RPE cell survival, or vascularization, and therefore it has been suggested that it plays a role in the pathophysiology of AMD 33.

### Endothelin I

Endothelin I is a protein expressed in the retina and choroid 67. It acts on two receptors located in smooth muscle cells, resulting in both vasoconstriction and vasodilation 68. It is secreted from the basal surface of the RPE, and by interacting with its receptors in the choriocapillaris, can regulate blood flow 27. It has been proposed that stresses involved in the breakdown of the blood–retinal barrier, as in AMD, can cause an increase in the expression and secretion of endothelin I, suggesting a role in wound repair *via* effects on proliferation and cell migration 27.

### Vascular endothelial growth factor

Vascular endothelial growth factor is a pro-angiogenic growth factor that is secreted preferentially from the basal surface of the RPE 12, 50. VEGF modulates and maintains the extracellular space in and around the Bruch's membrane, and modulates the growth/density of endothelial cells in the choriocapillaris 69–71. Its secretion is normally tightly regulated, thus preventing its concentration levels from surpassing a critical threshold able to induce vascularization 16. However, considering its potent angiogenic properties, it is plausible that the incorrect localization of this growth factor can be a critical factor in late-stage exudative AMD. This theory is supported by findings that elevated local concentration of VEGF can result in the formation of abnormal blood vessels, as seen in AMD 72.

### Cystatin C

Cystatin C is a reversible inhibitor of cysteine proteinases including papain and cathepsins B, H, L and S 73, 74. A polymorphism present in the genomic sequence of the cystatin C gene produces a mutant variant of the protein referred to as variant B. Homozygosity for this variant has been shown to correlate with an increased risk of developing exudative AMD, with a relatively early onset 26. It is likely that cystatin C is secreted from the basolateral side of the RPE 25 and the AMD-related variant has been shown to present a significantly reduced secretion. When considering this protein's function, its link to AMD, and the fact that the activity of its main group of substrates, the cathepsins, is altered in AMD 75–77, it is conceivable that its directional secretion could be highly important in the maintenance and turnover of the Bruch's membrane and choriocapillaris.

## Mechanisms of protein secretion/polarized secretion

A large number of soluble proteins are secreted from the PM *via* the relatively well-characterized ‘classical’ secretion pathway. These proteins contain N-terminally located signal peptides, directing them co-translationally to the translocation apparatus of the ER 78. Following vesicular transport from the ER to the Golgi apparatus, secreted proteins are then packaged into Golgi-derived vesicles that ultimately fuse with the PM, releasing their contents into the extracellular space 79–82. Alternatively, many proteins are also secreted from the cell *via* other, ‘non-classical’ mechanisms. These proteins typically lack a signal peptide, and are excluded from organelles that are essential during ‘classical’ secretion, such as the ER and Golgi. These mechanisms of secretion are reviewed in detail elsewhere 83, and include import into endosomal sub-compartments, direct translocation across the PM, and membrane blebbing, releasing the secreted proteins *via* exosomes.

**Fig. 2:**
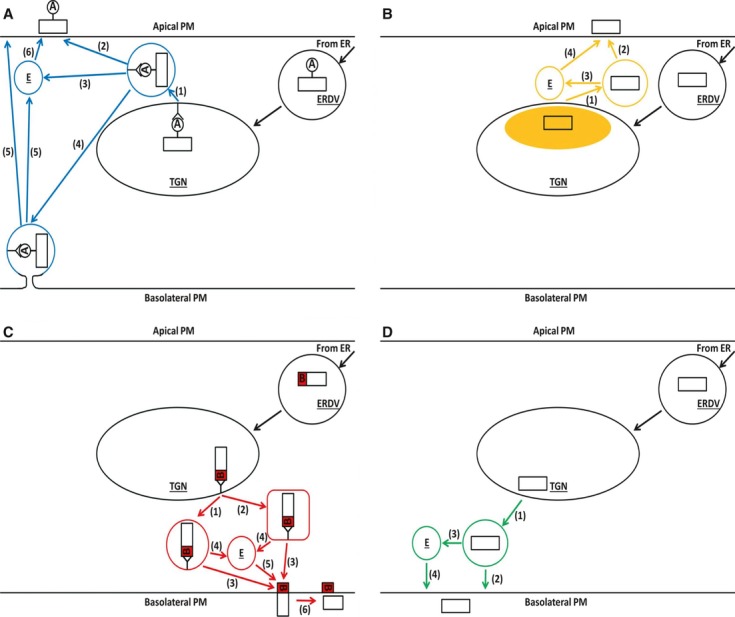
Models for targeted protein secretion from the endoplasmic reticulum (ER)-Golgi secretory pathway. Proteins containing apical sorting signals (A in white circle), basolateral sorting signals (B in red square) or no sorting signals (plain white rectangle) are delivered from the ER in ER derived vesicles (ERDV). Upon entering the Golgi apparatus, proteins progress to the trans-Golgi network (TGN) and sorting occurs. In pathway (**A**), apical signals (glycans, GPI linkages) interact with specific cellular machinery (*e.g*. apical targeting receptors, lipid rafts), resulting in packaging into apical vesicles (1). These vesicles can then traffic directly to the apical plasma membrane (PM) for extracellular release (2). Alternatively, some proteins are first trafficked to endosomes (E) (3), or to the basolateral PM (4). Basally trafficked proteins are subsequently redirected to the apical PM *via* transcytosis, either directly, or *via* endosomes (5). Proteins in the endosomal pathway are subsequently trafficked to the apical PM (6). In pathway (**B**), proteins that lack a typical targeting signal can undergo ‘specialized packaging’ in particular environments (yellow area). For example, proteins with an affinity for lipids can localize in areas of high lipid content. These proteins are subsequently packaged into vesicles and targeted to the apical PM directly (1, 2), or indirectly *via* endosomes (1, 3, 4). In pathway (**C**), basolateral signals interact with a specific cellular machinery (*e.g*. clathrin adapters), resulting in packaging into basolateral vesicles (1). It is possible that some secreted proteins are packaged into secretory vesicles (2) distinct from those used for transmembrane proteins (circular vesicle *versus* rectangular). Basolateral vesicles can then traffic to the basolateral PM directly (3), or *via* endosomes (4, 5). For some secreted proteins (*e.g*. TGFα), the cytoplasmic domain is cleaved, resulting in secretion of the mature form (6). Pathway (**D**), can act as a default for any proteins not otherwise targeted (proteins that lack a typical targeting signal), or alternatively, proteins that do not enter pathway (**B**) (*e.g*. due to lack of affinity for lipids). These proteins are subsequently packaged into vesicles and targeted to the basolateral PM directly (1, 2), or indirectly *via* endosomes (1, 3, 4).

In polarized cells, secretion requires an extra level of complexity and control to ensure certain proteins are targeted to, and released from the appropriate cell surface. Mechanisms required for such control have been studied extensively within the ‘classical’ secretion pathway, and have been reviewed in detail elsewhere 84–86. Although the majority of this work has focused on the targeting of membrane-bound proteins, many secreted proteins are likely targeted *via* similar mechanisms. Thus, the basolateral *versus* apical sorting mechanisms in the secretory pathway is believed to rely on complex membrane trafficking pathways which also underpin the distinct specialization of the apical and basolateral PM domains. Targeting of proteins to a particular cell surface takes place at the level of the trans-Golgi network (TGN), following incorporation of apical and basolateral proteins into distinct vesicles 87–90, a process that usually requires the presence of directional sorting signals. More recently, it has been suggested that some targeting may actually occur earlier than the TGN 91. In addition, the endocytosis pathway itself is regulated to preserve the polarity of lipid and protein components following internalization and recycling. Some of the potential mechanisms required for directional protein secretion are discussed below and summarized in Figure 2.

### Basolateral sorting signals

Basolateral sorting signals typically consist of tyrosine-based (YxxØ, FxNPxY) or di-leucine-based peptide sequences, found in the disposed, cytoplasmic portion of transmembrane proteins 84, 89, 91. Interestingly, these basolateral signals are usually dominant over their apical counterparts 92. In many cases, these signal peptides are recognized, and directly bind to heterotetrameric clathrin adapter protein complexes. This event triggers the incorporation of cargo into nascent vesicles, which are subsequently trafficked to the basolateral PM 84, 93. As these adaptor proteins are thought to mediate transport between the TGN and endosomal compartments, it is possible that some basolateral proteins traverse recycling endosomes before reaching the PM 91. Indeed, the importance of indirect sorting of proteins *via* such endosomal pathways has been highlighted 94–96. Alternatively, some proteins are targeted directly to the PM 97. Although there are only few examples of basolateral signals demonstrated in secreted proteins, it is likely that these are sorted in a similar fashion as transmembrane proteins.

Transforming growth factor-α (TGFα) provides one of the relatively few examples of a typical basolateral signal in a secreted protein. Its precursor form (pro-TGFα) contains a dominant basolateral sorting signal within the cytoplasmic region that is subsequently cleaved to produce the soluble, mature form 98. It has also been suggested that basolateral secretion can occur independently of any sorting signal, relying instead on a default, cell-dependent pathway that is governed by the association of particular proteins to intracellular lipids 99. The overall lipid content of the cell, as well as the lipid composition of apical and basal vesicles are therefore important factors in this process. There is also evidence of separate branches within the basolateral sorting pathway for secreted and transmembrane proteins, resulting in loading into distinct carrier vesicles 100.

### Apical sorting signals

Apical sorting signals are much more diverse than basolateral signals and consist of post-translational modifications, rather than distinct peptide sequences. Perhaps the most extensively studied of these are N- and O-linked glycans, which are present on particular proteins. Two models have been proposed to explain how these glycans are involved in apical sorting 101. The first suggests that these carbohydrates are critical for the assumption of a correct conformation that is necessary to progress along the biosynthetic pathway. The second model suggests the existence of a sorting receptor that recognizes carbohydrates, or alternatively, protein conformations that are dependent upon such carbohydrates. Glycans can also cause aggregation of proteins into pre-export complexes 91. Non-glycan apical sorting signals have also been suggested, imagined as three-dimensional proteinaceous patches 101. Glycosylphosphatidylinositol (GPI) linkages enable sorting through association with apical PM-bound lipid rafts 92. Apical sorting can also occur independently of signals (glycosylation). In this case, it is hypothesized that certain cell types can provide an environment that encourages ‘specialized packaging’ of proteins destined for apical secretion 102.

The route that apical proteins take to the PM seems as equally diverse as the signals that direct them. Many transmembrane proteins destined for the apical PM are transported from the TGN in different vesicles, and are then processed *via* distinct subsets of endosomal compartments before reaching their final destination 103, 104. It is, however, hypothesized that soluble, secreted proteins may in fact be targeted directly to the PM, to avoid degradation within the endocytic pathway 86. RPE cells, in particular, also differ in their processing of many apical proteins, directing them first to the basolateral PM, before a process of relocation to the apical PM *via* transcytosis 105.

Mechanisms for targeting secreted proteins for apical PM release are similar to those for transmembrane proteins. Glycans can promote such targeting, as is the case during apical sorting of rat growth hormone 106. Glycoprotein 2 is targeted to the apical PM *via* a GPI anchor in its transmembrane domain. Subsequent cleavage then results in secretion of the soluble form 107 in a similar manner to the basolateral secretion of TGFα. Human growth hormone, thyroglobulin and parathyroid hormone have been shown to be secreted apically, independently of known apical signals, relying instead on particular cellular conditions 102.

### Consequences of impaired RPE protein secretion

It is becoming increasingly apparent that directional protein secretion is a highly regulated and complex process, and that malfunctions occurring at any step in these pathways could lead either to increased/decreased levels of essential growth factors and structural proteins in the extracellular space or to their incorrect intracellular/extracellular localization. It is therefore possible that such malfunctions could serve as relatively overlooked mechanisms in the pathophysiology of the highly secretory RPE, with specific consequences for the development of retinal disorders such as AMD.

Indeed, such alterations in protein secretion have been implicated in the development of other diseases. Such an example is the increase in growth factor secretion from cancer cells, leading to autocrine growth stimulation and metastasis 108. Secretion of cystatin C variant B from human primary fibroblasts has also been shown to be reduced compared with the wild-type 109. It is possible that such reduced secretion of cystatin C in the brain can result in a lack of protection following toxic insult, or in stem-cell mediated regeneration, factors that may contribute to the development of Alzheimer's disease 109. A recent study has demonstrated that cells derived from patients with AMD display a striking difference in the secretion of several proteins compared with matched healthy donors. Many of these proteins are involved in angiogenesis and protein aggregation, processes that have been heavily implicated in AMD pathogenesis 110.

Extracellular mislocalization of secreted proteins can occur as a result of changed cell polarity and alterations in directional secretion. Such alterations can also have implications in disease development. The presence of the Alzheimer's-linked, ‘Swedish’ double mutation in the amyloid precursor protein results in a proportion being mis-secreted from the apical, rather than basolateral PM 111. Directional secretion can also be altered as a result of external stimulation of cells with cytokines 112, 113, the activity of which can be altered in disease states. Total secretion of important factors such as PEDF (apically or basally) from RPE cells can be dependent on the cell attaining a highly polarized configuration 16, 114, again highlighting the importance of polarity for correct protein secretion.

Some of the RPE-secreted proteins highlighted above have been shown to have altered secretion patterns during AMD development. MMP-2 is secreted at levels threefold higher in AMD RPE cells compared with healthy RPE 110. It is possible that this protease is indirectly involved in angiogenesis *via* its proteolytic and ECM remodelling properties 115. PEDF secretion is also, surprisingly, increased in AMD RPE cells 110. It has been suggested that this may be a compensatory response by the RPE to balance the angiogenic properties of VEGF 110. This same study 110 was also able to show an increase in clusterin secretion and a decrease in SPARC secretion in AMD cells. The precise function of clusterin has not been defined, but its presence in Drusen suggests that it may be involved in their formation 116, 117, therefore contributing to one of the greatest hallmarks of AMD. SPARC is known to have anti-angiogenic properties 118, 119, meaning reduced levels in the choroid could aid in neovascularization.

One of the most abundantly expressed and secreted proteins of the RPE is the cysteine proteinase inhibitor cystatin C 120. The role that this protein plays in the RPE has not yet been characterized, yet its function as a cysteine proteinase inhibitor together with its extracellular targeting 25 suggest an important involvement in matrix remodelling and turnover, processes that are essential for retinal homoeostasis. A signal peptide present in the 26 amino acid leader sequence 121 of cystatin C targets it to the ‘classical’ ER/Golgi secretory pathway 25. The polymorphism that results in the variant B cystatin C translates into an amino acid substitution (alanine to threonine) at the penultimate position of this leader sequence. This alteration results in a failure of cystatin C to efficiently enter the secretory pathway, leading to a diffuse intracellular distribution of the protein, an association with mitochondria, and a reduction in its secretion of ca. 50% 122, 123. Reduced hydrophobicity of the signal sequence caused by the amino acid substitution is thought to be the cause of this secretory malfunction 124.

## Concluding remarks

The tissues on either side of the RPE present two different microenvironments, each placing varying demands on the RPE in terms of protein requirements. High levels of particular growth factors and structural proteins that can be beneficial in one compartment can be detrimental in the other. Maintaining the correct concentrations of particular factors, in the correct location, at the correct time is therefore of critical importance for retinal health.

This article highlights several examples of directional protein secretion by the RPE, all of which could play important roles in maintaining its surrounding extracellular environments. Malfunctions in trafficking/secretory pathways can lead to mis-localization of these proteins, which can ultimately manifest as a number of AMD symptoms.

Therapies such as RPE transplants and gene therapy could offer improved treatments for AMD in the future. The importance of polarity and directional protein secretion in maintaining RPE/retinal functioning will be key considerations in the further development of such therapies.
